# Epidemiological characteristics of imported respiratory infectious diseases in China, 2014‒2018

**DOI:** 10.1186/s40249-022-00944-6

**Published:** 2022-03-04

**Authors:** Jin-Long Wang, Tao Chen, Le-Le Deng, Ya-Jun Han, Da-Yan Wang, Li-Ping Wang, Guang-Xue He

**Affiliations:** 1 National Institute for Viral Disease Control and Prevention, Chinese Center for Disease Control and Prevention, Beijing, China; 2 Division of Infectious Diseases, Chinese Center for Disease Control and Prevention, Beijing, China

**Keywords:** Imported respiratory infectious disease, Influenza, Epidemiological characteristics, Associated factors, China

## Abstract

**Background:**

With the progress of globalization, international mobility increases, greatly facilitating cross-border transmission of respiratory infectious diseases (RIDs). This study aimed to analyze the epidemiological characteristics and factors influencing imported RIDs, with the goal of providing evidence to support adoption of high-tech, intelligent methods to early find imported RIDs and prevent their spread in China.

**Methods:**

We obtained data of imported RIDs cases from 2014 to 2018 from the Inbound Sentinel Network of Customs and the National Notifiable Diseases Reporting System in China. We analyzed spatial, temporal, and population distribution characteristics of the imported RIDs. We developed an index to describe seasonality. Pearson correlation coefficients were used to examine associations between independent variables and imported cases. Data analyses and visualizations were conducted with R software.

**Results:**

From a total of 1 409 265 253 inbound travelers, 31 732 (2.25/100 000) imported RIDs cases were reported. RIDs cases were imported from 142 countries and five continents. The incidence of imported RIDs was nearly 5 times higher in 2018 (2.81/100 000) than in 2014 (0.58/100 000). Among foreigners, incidence rates were higher among males (5.32/100 000), 0–14-year-olds (15.15/100 000), and cases originating in Oceania (11.10/100 000). The vast majority (90.3%) of imported RIDs were influenza, with seasonality consistent with annual seasonality of influenza. The spatial distribution of imported RIDs was different between Chinese citizens and foreigners. Increases in inbound travel volume and the number of influenza cases in source countries were associated with the number of imported RIDs.

**Conclusions:**

Our study documented importation of RIDs into China from 142 countries. Inbound travel poses a significant risks bringing important RIDs to China. It is urgent to strengthen surveillance at customs of inbound travelers and establish an intelligent surveillance and early warning system to prevent importation of RIDs to China for preventing further spread within China.

**Graphical Abstract:**

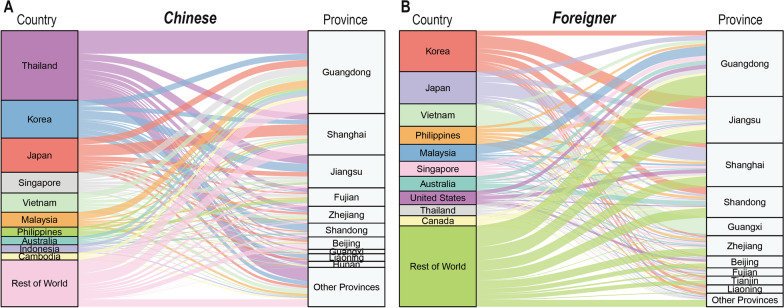

## Background

Globalization, increasing air travel, and greater population mobility facilitate inter-regional spread of infectious diseases, including emerging infectious diseases (EIDs) [1, 2]. In 2018, there were 1.4 billion international trips, 10% of which involved Chinese citizens [3]. Increased population mobility leads to more rapid and higher volume cross-border spread of infectious diseases [1]. The 1918 influenza pandemic spread by sea and land, but its international spread was slower than cross-border spread of infectious diseases is today. Indeed, infectious diseases can traverse the globe within a single day [4–6]. More attention should be paid to surveillance, early warning, prevention, and control of cross-border spread of infectious diseases.

Since the twentieth century, EIDs, especially severe acute respiratory infectious diseases (RIDs), occur constantly and have had an enormous impact on human health and social development [2]. Rapid development of human societies, convenient transportation, and frequent international exchanges have accelerated cross-border spread of RIDs, posing significant challenges to their prevention and control. Middle East respiratory syndrome (MERS) was first reported in Saudi Arabia in 2012, and spread to other countries through travel of infectious MERS cases. As of April 26, 2016, 1728 laboratory-confirmed MERS cases had been reported in 27 countries [7]. In 2015, Guangdong Province reported their first imported MERS case—imported from the Republic of Korea—but Guangdong’s rapid response and control avoided onward spread and outbreaks of MERS [8, 9].

Over recent decades, several international studies evaluated the relationship between population mobility and spread of infectious diseases [10–12]. Two studies of travel-related infections, based on long-term surveillance data analysis, described the epidemiology of imported infections [13–15]. However, detailed epidemiological characteristics and influencing factors for imported RIDs in China have not been further explored and analyzed. To fill this knowledge gap, we investigated the epidemiological characteristics and factors related to imported RIDs in China, with the goal of promoting use of high-tech methods to rapidly detect and prevent importation of key RIDs to China.

## Methods

### Data collection and resources

We obtained imported RIDs surveillance data and inbound traveler data, reported between January 1, 2014 and December 31, 2018, from the entry–exit sentinel network of customs (EESNC), which consists of 272 quarantine sentry points in the 31 provinces of mainland China [13], and from the National Notifiable Disease Reporting System (NNDRS) of the Chinese Center for Disease Control and Prevention (CCDC) [16]. Imported RIDs cases were classified into Chinese citizen or foreigner based on documented nationality. We obtained data on 430 imported RIDs cases from 48 countries from NNDRS, and data on 31 302 imported RIDs cases from 140 countries from EESNC. Analyzed variables included gender, age, nationality, disease, time of importation, country of origin, province, entrance way, travel purpose, detection method, and main symptoms. Imported RIDs case data from the EESNC and NNDRS are routine surveillance data that were used under license for our study; all case data were anonymized.

We downloaded global influenza weekly surveillance data of 174 countries for January 1, 2014, to December 31, 2018, from FluNet (https://www.who.int/tools/flunet) [17] to analyze the correlation between the number of reported influenza cases in source countries and the number of imported cases in China analyzing with data from EESNC and NNDRS.

Statistics on gender, age, and continent of origin of foreign nationals from 25 countries and regions traveling to China each year were obtained from the China Statistical Yearbook (http://www.stats.gov.cn/english/Statisticaldata/AnnualData) [18]. However, the incidence of imported RIDs from Chinese travelers by gender, age, and continent could not be calculated because this information was not available on inbound Chinese travelers.

### Data management and quality control

Two research staff unified criteria and definitions of the data elements in the data bases. Imported RIDs information came from linkage of EESNC and NNDRS; duplicates were removed. Because influenza accounts for more than 90% of the total imported RIDs, we evaluated the relation between imported influenza RIDs and region of origin. We made a scatter plot for the continents of origin using imported influenza data and annual influenza surveillance report data from source countries.

### Statistical analysis

Imported RIDs incidence per 100 000 travelers was estimated as the number of imported cases divided by the number of inbound travelers during a given time period. A seasonal index of imported RIDs was calculated by dividing the number of imported respiratory infections per month by the average number of imported RIDs per year. A seasonal index greater than 1 indicates that the number of imported RIDs in a given month was higher than the monthly average. We used a radar chart of the monthly seasonal index of imported RIDs to show seasonal distribution characteristics. Continuous variables were summarized as medians and ranges, and categorical variables were summarized as numbers and percentages. Pearson correlation coefficients were used to examine associations between independent variables and imported infections. Data analysis and visualization were conducted with R version 4.0.5 (https://www.r-project.org/), using tidyverse, reshape2, RColorBrewer, lubridate, and basic R packages.

## Results

### General characteristics

**Fig. 1:**
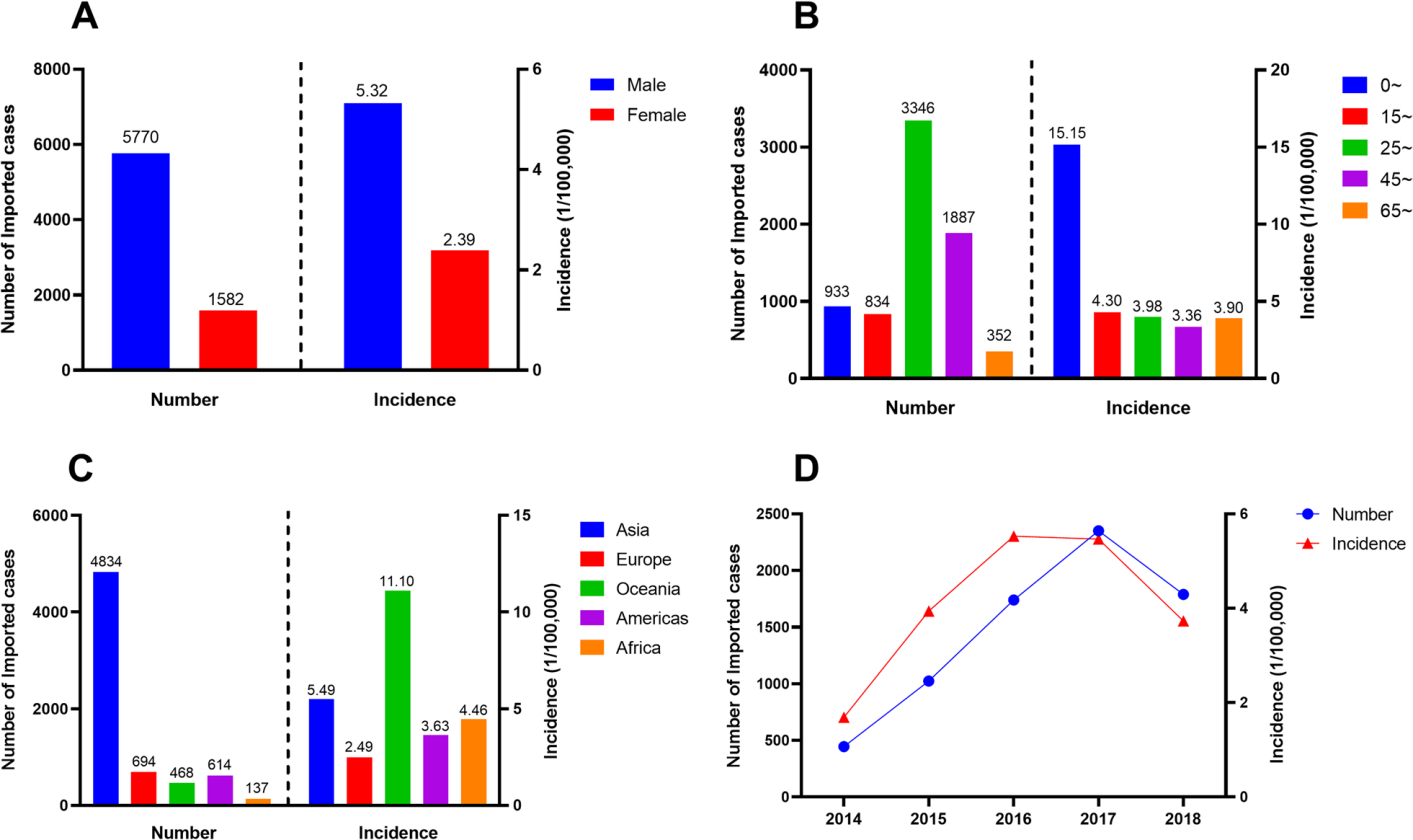
The number and incidence of imported respiratory infectious diseases among inbound foreigners by **A** gender, **B** age group, **C** continent, and **D** year

From 2014 to 2018, approximately 1409.27 million passengers entered mainland China by a variety of legal routes; 87.6% of the enterers were Chinese citizens and 12.4% were foreign nationals. During these 5 years, 31 732 imported RIDs cases were reported—an incidence of 2.25 RIDs cases per 100 000 international travelers entering China. The imported RID incidence in 2018 (2.81/100 000) was nearly 5 times that in 2014 (0.58/100 000). The imported RID incidence among foreigners (4.21/100 000) was higher than among Chinese citizens (1.97/100 000). Among foreigners, the incidence was highest in males (5.32/100 000), in 0‒14-year-olds (15.15/100 000), and in travelers from Oceania (11.10/100 000). The average age of the 20 758 (65.4% of RIDs) males importing RIDs was older than that of females importing RIDs [male median age: 34, interquartile range (IQR): 21‒48; female median age: 28, IQR: 11‒44]. Twenty-seven types of RIDs were detected, including influenza (90.3%), rhinovirus (3.2%), and others (6.5%) (Tables 1, Table 2, Fig. 1).

**Table 1:** Characteristics of 31 732 imported respiratory infectious diseases cases in China, 2014–2018

Variables	Total cases *N* (%)	Chinese *N* (%)	Foreigner *N* (%)
Sex			
Male	20 758 (65.4)	14 988 (61.5)	5770 (78.5)
Female	10 974 (34.6)	9392 (38.5)	1582 (21.5)
Age			
0~	7240 (22.8)	6307 (25.9)	933 (12.7)
15~	3535 (11.1)	2701 (11.1)	834 (11.3)
25~	12 033 (37.9)	8687 (35.6)	3346 (45.5)
45~	7360 (23.2)	5473 (22.5)	1887 (25.7)
65~	1564 (4.9)	1212 (5.0)	352 (4.8)
Disease			
Influenza	28 656 (90.3)	22 303 (91.5)	6353 (86.4)
Rhinovirus	1005 (3.2)	777 (3.2)	228 (3.1)
Others^a^	2071 (6.5)	1300 (5.3)	771 (10.5)
Year			
2014	1440 (4.5)	994 (4.1)	446 (6.1)
2015	3148 (9.9)	2123 (8.7)	1025 (13.9)
2016	5084 (16.0)	3344 (13.7)	1740 (23.7)
2017	13 233 (41.7)	10 882 (44.6)	2351 (32.0)
2018	8827 (27.8)	7037 (28.9)	1790 (24.4)
Source continent			
Africa	262 (0.8)	125 (0.5)	137 (1.9)
Americas	1091 (3.4)	477 (2.0)	614 (8.4)
Asia	16 749 (52.8)	11 915 (48.9)	4834 (65.8)
Europe	1528 (4.8)	834 (3.4)	694 (9.4)
Oceania	989 (3.1)	521 (2.1)	468 (6.4)
Unknown	11 113 (35.0)	10 508 (43.1)	605 (8.2)
Imported province			
Non-border province	3042 (9.6)	2592 (10.6)	450 (6.1)
Border province	28 645 (90.3)	21 746 (89.2)	6899 (93.8)
Unknown	45 (0.1)	42 (0.2)	3 (< 0.1)
Entrance way			
Air	15 251 (48.1)	12 160 (49.9)	3091 (42.0)
Land	9861 (31.1)	8961 (36.8)	900 (12.2)
Water	6020 (19.0)	3029 (12.4)	2991 (40.7)
Unknown	600 (1.9)	230 (0.9)	370 (5.0)
Purpose			
Tourism	10 356 (32.6)	9047 (37.1)	1309 (17.8)
Business	1834 (5.8)	1369 (5.6)	465 (6.3)
Labour	1351 (4.3)	560 (2.3)	791 (10.8)
Studying	293 (0.9)	156 (0.6)	137 (1.9)
Visiting friends or relatives	626 (2.0)	310 (1.3)	316 (4.3)
Sailor	1538 (4.9)	545 (2.2)	993 (13.5)
Others	1937 (6.1)	1051 (4.3)	886 (12.1)
Unknown	13 797 (43.5)	11 342 (46.5)	2455 (33.4)
Finding way			
Fever screening	23 997 (75.6)	19 832 (81.4)	4165 (56.7)
Medical inspection	5246 (16.5)	3286 (13.5)	1960 (26.7)
Selfdeclaration	561 (1.8)	419 (1.7)	142 (1.9)
Reported by onboard staff	1468 (4.6)	743 (3.1)	725 (9.9)
Others	460 (1.5)	100 (0.4)	360 (4.9)
Main symptom			
Cough	12 951 (40.8)	10 170 (41.7)	2781 (37.8)
Fever	7592 (23.9)	5226 (21.4)	2366 (32.2)
Headache	4556 (14.4)	3426 (14.1)	1130 (15.4)
Chills	3116 (9.8)	2210 (9.1)	906 (12.3)
Facial flushing	1836 (5.8)	1421 (5.8)	415 (5.6)
Muscle pain	1105 (3.5)	832 (3.4)	273 (3.7)

^a^Other respiratory diseases including: Pulmonary tuberculosis, pneumococcal infection, Mycoplasma pneumoniae infection, Legionnaires’ disease, Streptococcus infection, Chlamydia pneumoniae infection, Pertussis, Pneumococcal infection, Acute nodular pharyngitis, Adenovirus infection, Haemophilus influenzae infection, Respiratory syncytial virus infection, Chickenpox, Human metapneumovirus infection, coronavirus infection, parainfluenza virus infection, measles, mumps, bocavirus infection, rubella, scarlet fever, infectious atypical pneumonia, parvovirus infection, Middle East Respiratory Syndrome

**Table 2:** Incidence of imported respiratory infectious diseases in China, 2014–2018

Year	Chinese			Foreigner			Total		
	Case (*n*)	Passenger (*N*)	Incidence (1/100 000)	Case (*n*)	Passenger (*N*)	Incidence (1/100 000)	Case (*n*)	Passenger (*N*)	Incidence (1/100 000)
2014	994	222 879 100	0.45	446	26 360 800	1.69	1440	249 239 900	0.58
2015	2123	242 972 187	0.87	1025	25 985 400	3.94	3148	268 957 587	1.17
2016	3344	251 216 040	1.33	1740	31 483 700	5.53	5084	282 699 740	1.80
2017	10 882	250 888 523	4.34	2351	42 943 000	5.47	13 233	293 831 523	4.50
2018	7037	266 585 503	2.64	1790	47 951 000	3.73	8827	314 536 503	2.81
Total	24 380	1 234 541 353	1.97	7352	174 723 900	4.21	31 732	1 409 265 253	2.25

### Spatial distribution

**Fig. 2:**
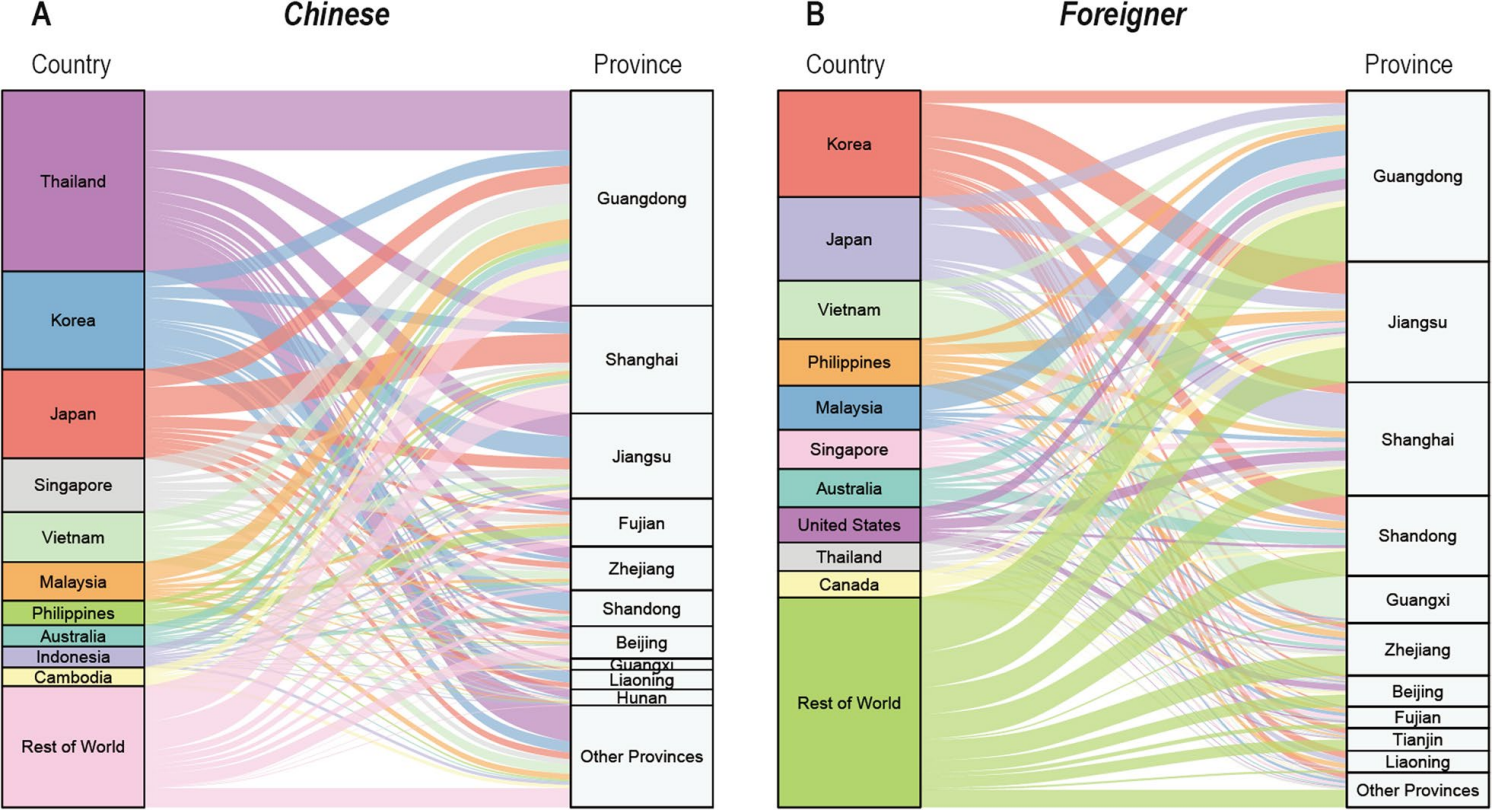
Spatial distribution of original countries and imported provinces in (**A**) Chinese and (**B**) foreigners

RIDs were imported from 142 countries and five continents, mostly Asian (52.8%), with entry along the developed coastal border areas of China. Most RIDs imported by Chinese citizens were imported from Thailand (14.1%), the Republic of Korea (7.9%), Japan (7.2%), and Singapore (4.2%). Most RIDs imported by foreign nationals were imported from the Republic of Korea (12.8%), Japan (10.4%), Vietnam (7.6%), and the Philippines (5.9%). Guangdong Province had the highest number of imported cases, followed by Jiangsu Province, and Shanghai (Figs. 1, 2).

### Temporal distribution

**Fig. 3:**
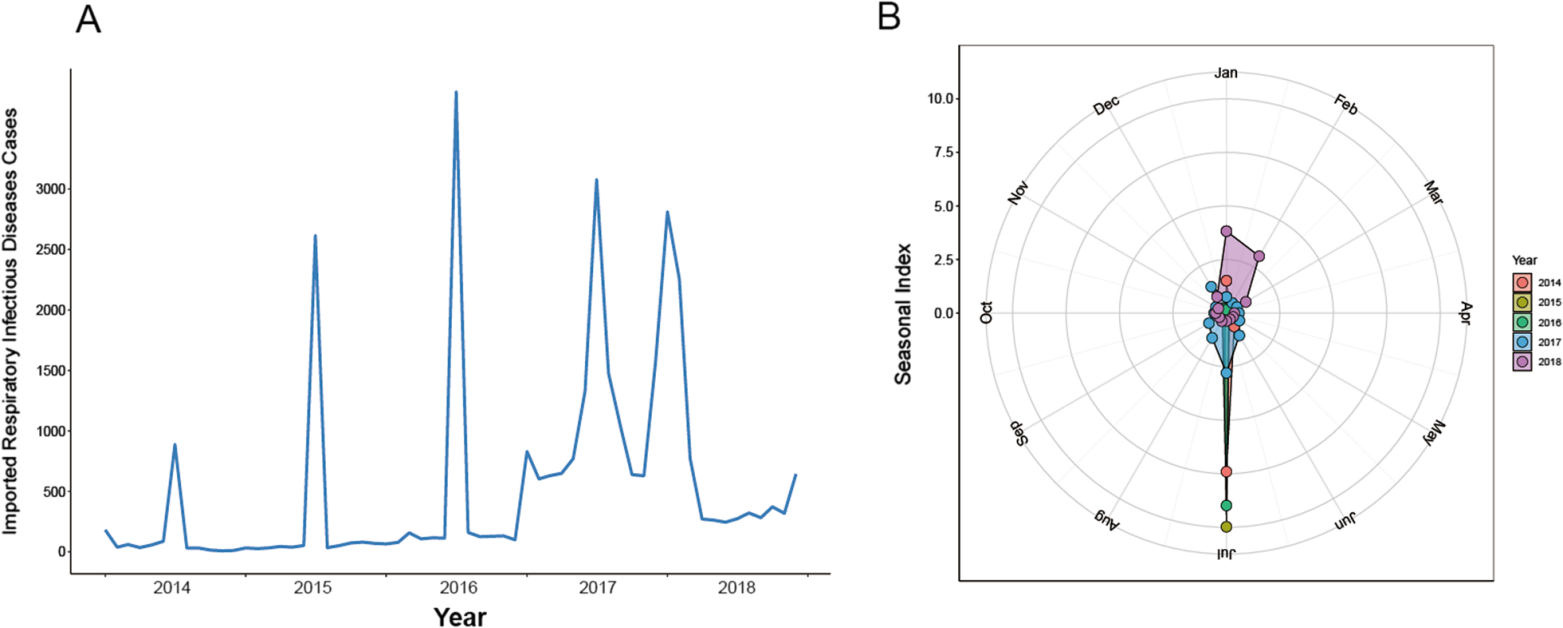
Temporal distribution of imported respiratory infectious diseases from 2014 to 2018.** A** Change of the number of imported RIDs by month from 2014 to 2018.** B** Change of the seasonal indices of imported RIDs by month from 2014 to 2018

From 2014 to 2017, the month with the most imported RIDs was July, with seasonal indices ranging from 2.79 to 9.97. In contrast, in 2018, the months with most imported RIDs were January–March (Fig. 3).

### Correlation analysis

**Fig. 4:**
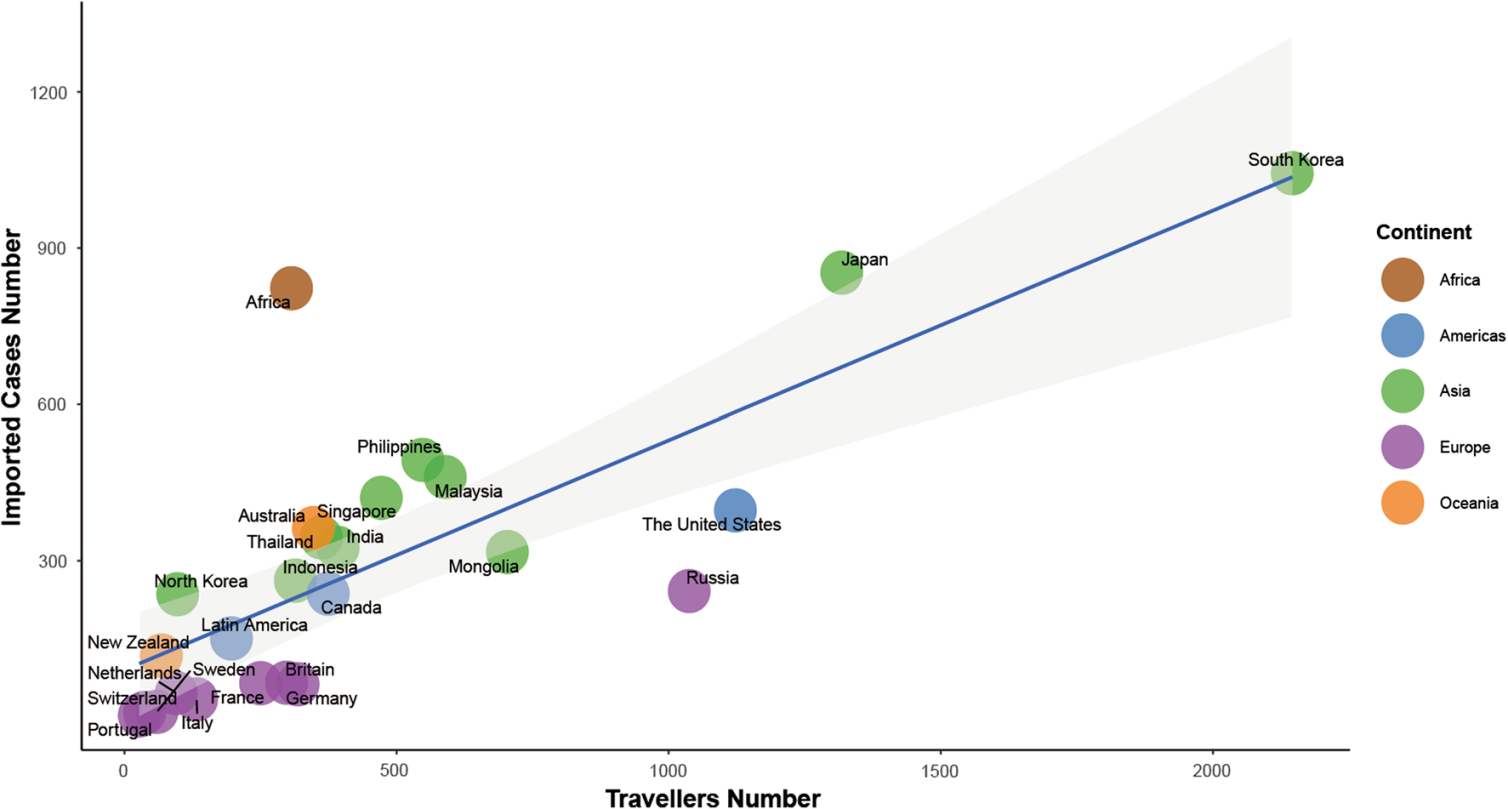
The relationship between the number of imported influenza cases and the number of inbound travelers among foreigners

The Pearson correlation coefficient between the number of imported cases and inbound travel volume was 0.775 [95% confidence interval (*CI*) 0.547 to 0.896]. A linear relationship was observed from the scatter plot, and a best-fit line was calculated using the least-squares method. By simple linear regression, the slope of the regression line was 0.441, implying that the average annual increase of imported RIDs cases was 0.441 per 10 000 foreigners entering China. The simple linear regression model explained 58.3% of the variance (adjusted R^2^ = 0.583); the regression model was statistically significant, F(1,23) = 34.5, *P* < 0.001 (Fig. 4).

**Fig. 5:**
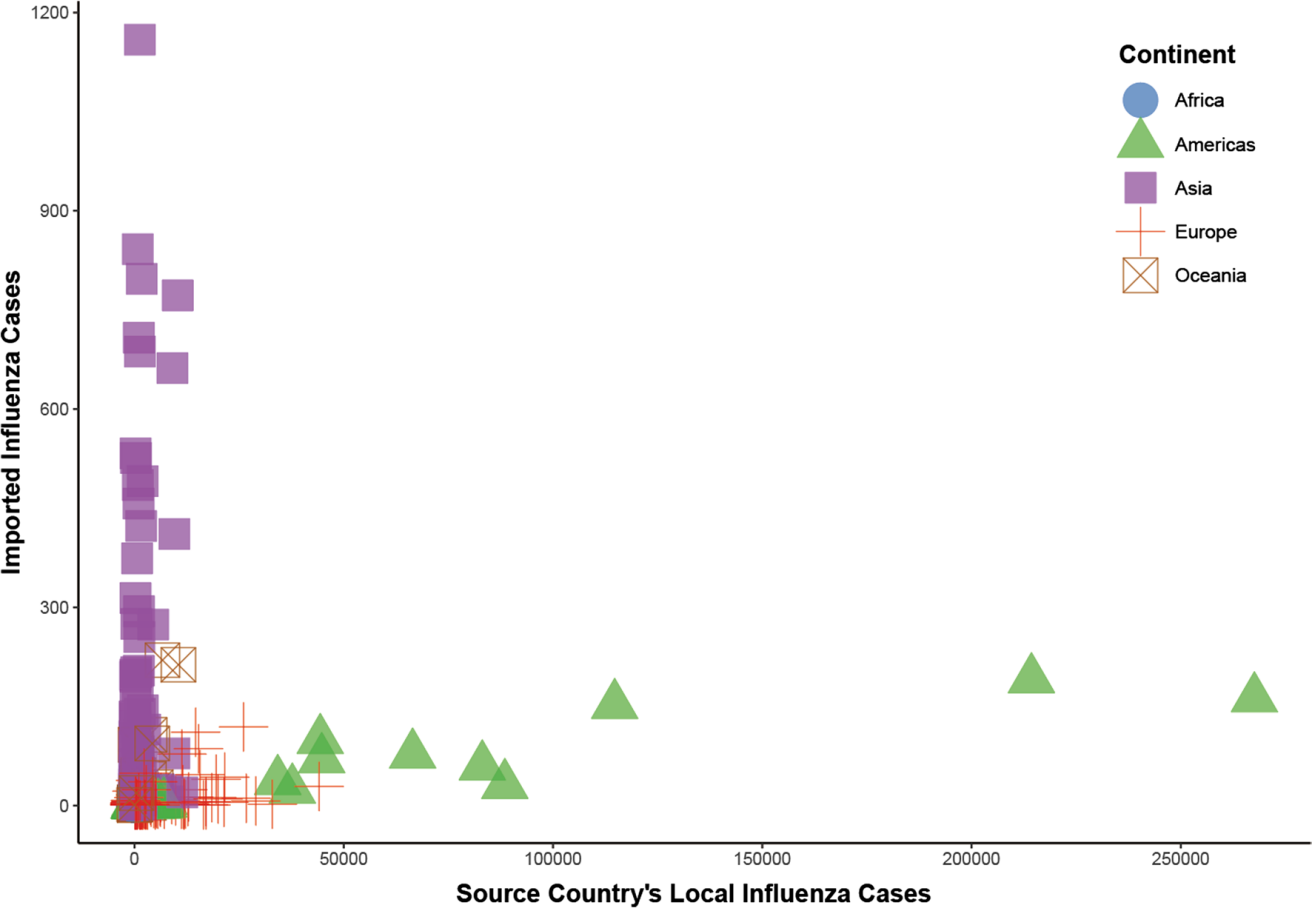
Scatter plot of the number of influenza cases in the original countries and the number of imported influenza cases

The number of imported influenza cases increased with increases of influenza reports from source countries. Importation epidemiologic characteristics varied by continent. Asian countries imported the most influenza cases to China, but the number of influenza cases reported by Asian countries was relatively low (Fig. 5).

## Discussion

Respiratory infectious diseases have a huge impact on the health of people around the world. With increasing globalization, social, economic, cultural, and population exchanges across countries worldwide are increasing, and the prevention and control of RID cross-border transmission is facing significant challenges [19, 20]. We described the epidemiological characteristics of imported RIDs to China during 2014–2018, and showed that number of imported RIDs cases was positively correlated with the number of inbound travelers and the number of influenza cases in source countries. Influenza accounted for the majority of imported RIDs and showed seasonality that was consistent with influenza seasonality. The spatial distribution of imported RIDs was different between Chinese citizens and foreign nationals. Among inbound foreigners, the incidence of imported RIDs was higher in males, 0–14-year-olds, and people from Oceania. The number of imported RIDs increased with increasing inbound travel volume and increasing number of influenza cases in source countries. We believe the findings can help improve surveillance and early warning of imported RIDs, promote joint prevention and control of infectious diseases among countries with a high burden of RIDs, and protect the health and safety of people around the world.

Other researchers have found differences in the incidence of travel-related infectious diseases by gender and age group [21, 22]. The differences are related to type of infectious disease, population characteristics, and destination of travel [22]. In our study, males and 0–14 year-olds were at greater risk of importing RIDs, consistent with previous studies [23–26]. Most travelers are male and may be more susceptible to RIDs due to risk behaviors and habits during travel. Children may be more vulnerable to respiratory infections because of a lack of adequate protection from infectious diseases and lower immunity than adults [27]. Most children have been infected with at least one influenza virus by the age of six [28]. Children infected with influenza during travel will increase the risk of infection in their parents and other relatives. Influenza risk can be reduced by vaccination in advance of travel [29].

We observed seasonal fluctuation of imported RIDs, and such seasonality may be affected by several factors—for example, international travel on holidays and seasonality of RIDs in originating areas [30]. There is more international travel during holidays. The Chinese traditional Spring Festival in January and February and summer vacation for Chinese students abroad from July to August lead to large, temporary increases in international travel that can promote cross-border spread of RIDs. Influenza accounted for the majority of imported RIDs, and the seasonality of influenza imported to China was similar to influenza seasonality in Asian source countries. RIDs import peaked in January–March 2018, which was different timing than from 2014–2017. This unusual timing may have been related to the 2018 spring influenza pandemic in northern hemisphere countries [31].

Travel volume is a key factor influencing the number of imported RIDs cases [32, 33]. Passengers who contracted an infectious disease before or during travel spread the infectious diseases to another country by cross-border travel. Generally, the risk of imported infectious diseases increases with increasing passenger volume. A previous study of the cross-border transmission of H1N1 showed a significantly increased risk of importing H1N1 from countries that received more than 1400 passengers from endemic countries [34]. Travel volumes, especially air travel volume data, are often used as an important variable for estimating the risk of infectious disease importation under certain conditions [35]. It has been reported that scientific travel restrictions can effectively curb the transboundary spread of infectious diseases in the emerging and reemerging infectious diseases outbreaks—a phenomenon that has been confirmed by the practice of combating infectious diseases in recent years [36–39].

The number of imported cases is associated with the number of reported cases in source countries, which is likely related to the prevalence of the disease and population of the source country [40]. In general, the higher the prevalence of infectious diseases in a source country, the higher risk of importation into neighboring countries. However, to assess risk of importing cases, a comprehensive analysis of the prevalence of RIDs in the importing countries is required, taking into account factors such as travel restrictions, cultural practices, social environment, distance traveled, transportation, and purpose of travel. We found that the average number of reported influenza cases in Asian countries of origin was lower than those in the Americas and Europe, but Asia had the highest number of imported cases. Travel volume variables can explain some of the variation, but some factors affecting the importation of infectious diseases require further study.

In order to assess and predict the risk of importation, investigators have developed statistical models based on data related to importation, such as international flights information, the epidemiology of selected diseases, and demographic information of source countries [41, 42]. Given the preconditions of these models, it is usually assumed that all residents have the same chance of infection and that all infected people have the same chance of boarding an outbound flight. However, this assumption may be unrealistic, leading to observed differences between predicted results of a model and the actual situation. Therefore, additional factors influencing imported infectious diseases should be further studied and incorporated into predictive model analyses.

We believe that there are additional factors affecting the epidemiology of imported RIDs that are in need of further exploration. For example, vaccination status influences imported RIDs. Environmental changes in temperature, humidity, air pollution, and sun exposure may influence RIDs spread [43]. Travel duration is often considered as a critical factor of imported RIDs. Since the data used in our study were mainly from EESNC and NNIDRS, it is possible to lose some information in our study and might lead to inexactly calculated incidence of imported RIDs.

## Conclusions

Imported respiratory infectious diseases incidence increased from 2014 to 2018. Gender, age, continent, inbound passenger volume, and the number of reported cases in source countries were associated with the incidence of imported RIDs. With annual increases in international travel, the potential risk of RIDs spread to China rises accordingly. Therefore, it is urgent to strengthen surveillance at customs for inbound travelers and establish an intelligent surveillance and warning system for imported RIDs to early find and prevent the RIDs from spreading to China. The health and safety of people around the world will be more effectively protected if infectious diseases are controlled in their countries of origin. It is therefore vital for the international community to support prevention and control of important RIDs for countries having limited resources.

## Data Availability

According to the requirement of the EESNC and NNDRS, the original data can be used only by our researchers and cannot be provided to others.

## Abbreviations

EIDs: Emerging infectious diseases
RIDs: Respiratory infectious diseases
MERS: Middle East respiratory syndrome
EESNC: Entry–exit sentinel network of customs
NNDRS: National Notifiable Disease Reporting System
CCDC: Chinese Center for Disease Control and Prevention
IQR: Interquartile range
*CI*: Confidence interval
